# Safety of adenosine stress perfusion cardiac magnetic resonance imaging in patients with aortic stenosis

**DOI:** 10.1186/1532-429X-13-S1-O38

**Published:** 2011-02-02

**Authors:** Stephen Darty

**Affiliations:** 1 Duke Cardiovascular Magnetic Resonance Center, Durham, NC, USA

## Background

Aortic stenosis (AS) is a relative contraindication to exercise, but the safety of adenosine stress perfusion cardiac magnetic resonance imaging (CMR) in patients with AS is unknown. The primary objective of this study was to determine the safety of adenosine perfusion CMR in patients with AS.

## Methods

Consecutive patients with known or suspected coronary artery disease (CAD) and native aortic valve stenosis (aortic valve area [AVA] <2 cm^2^ by planimetry), undergoing adenosine perfusion CMR were eligible for enrollment. CMR imaging included cine, first-pass perfusion at stress and rest, and delayed-enhancement imaging. CMR images were interpreted for CAD using an algorithm previously described by our group (Klem JACC 2006;47:1630-8).

## Results

**Figure 1:**
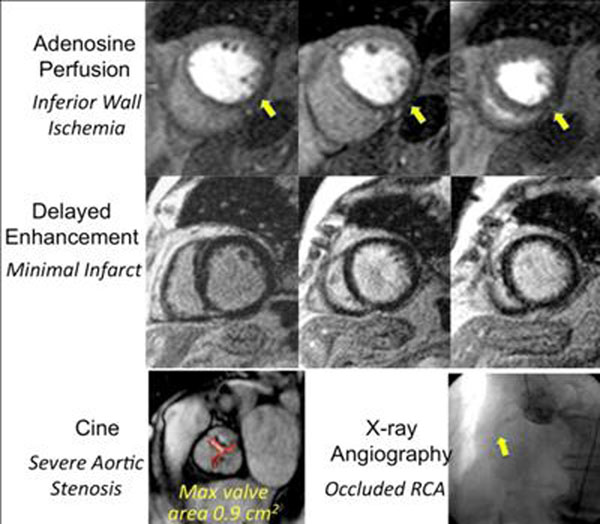


112 patients with mean age 76+11 yrs (52% female) were enrolled. Over all, 16%, 42%, and 41% has severe (AVA <1 cm^2^), moderate (AVA 1-1.5 cm^2^), and mild (AVA >1.5 cm^2^) AS. A mean dose of 31+10 mg adenosine was infused over 2.8+0.5 minutes. There were only 2 adverse events, both of which were transient. In the first patient (AVA=1.0 cm^2^) there was a sinus pause of 3 seconds, and the stress protocol and imaging were completed. In the second patient (AVA=1.1 cm^2^), there was prolonged chest pain with bradycardia and hypotension requiring discontinuation of adenosine. Symptoms resolved within 5 minutes without any further intervention. In both patients, pre and post stress ECG’s were unchanged. Diagnostic examinations were completed in 111 of 112 patients. Based on these results and using the exact method we estimate the 95% confidence intervals (CI) for the occurrence of a severe adverse event is 0-2.6% and transient (non-severe) event is 0.3-5.5%. Figure 1 shows an example of a positive stress perfusion study in a patient with severe AS (AVA=0.9 cm^2^). 57 of 111 patients (51%) had stress CMR studies indicative of significant CAD.

## Conclusions

Adenosine perfusion CMR is a safe alternative for non-invasive stress testing in patients with mild to severe AS.

